# Supplementary figures and images for: A multiomics analysis of direct interkingdom dynamics between influenza A virus and Streptococcus pneumoniae uncovers host-independent changes to bacterial virulence fitness
Source: PLoS Pathog. 2022 Dec 21;18(12):e1011020. doi: 10.1371/journal.ppat.1011020 (PMC9815659; doi:10.1371/journal.ppat.1011020)

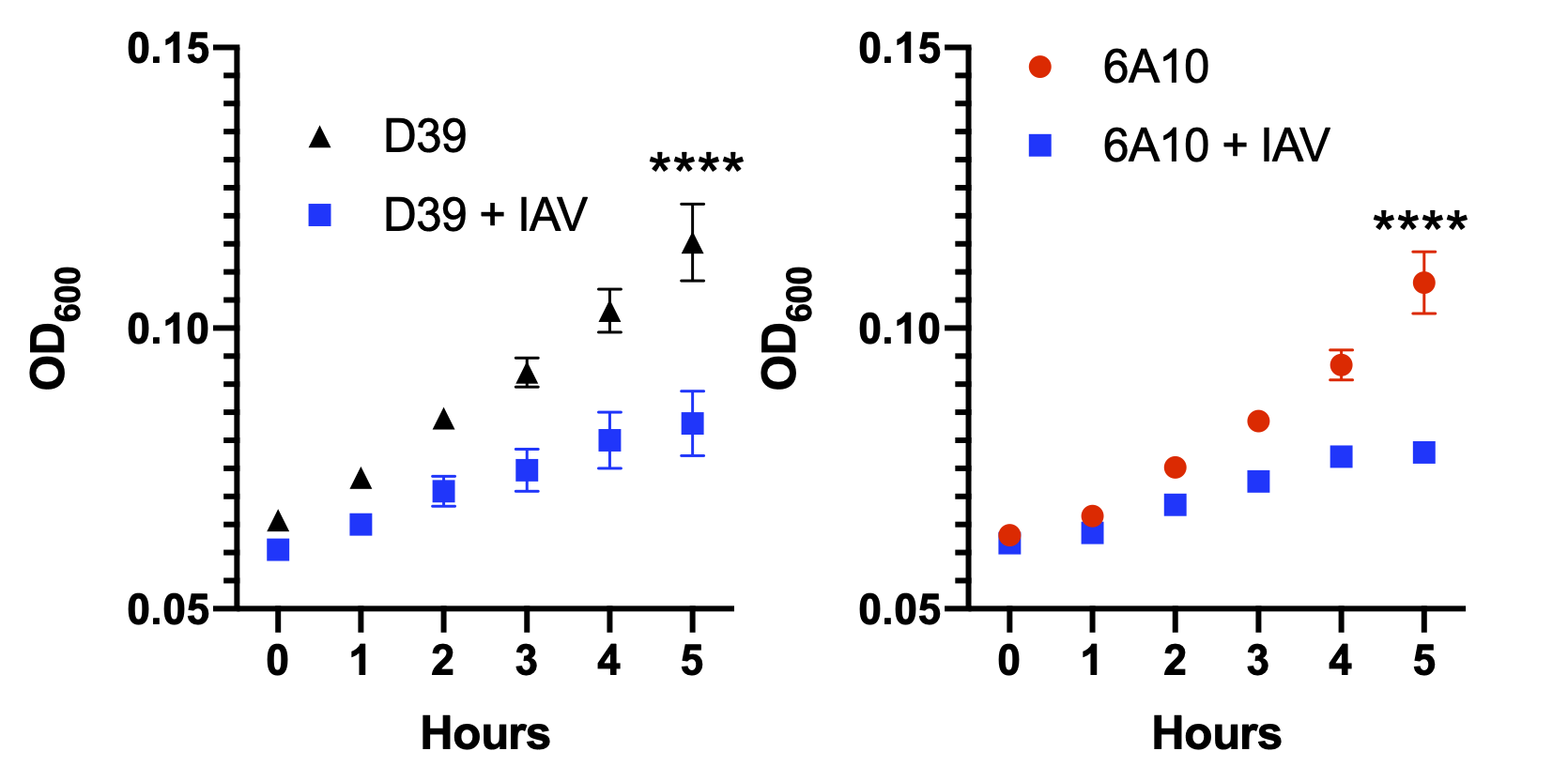

Supplement: S1 Fig — Growth curve of Spn strains D39 and 6A10 in liquid media with/without IAV. Data points represent mean +/- SD (two-way ANOVA, **p<0.05, ***p<0.0001 at t = 5h). (TIFF) [file ppat.1011020.s001.tiff]

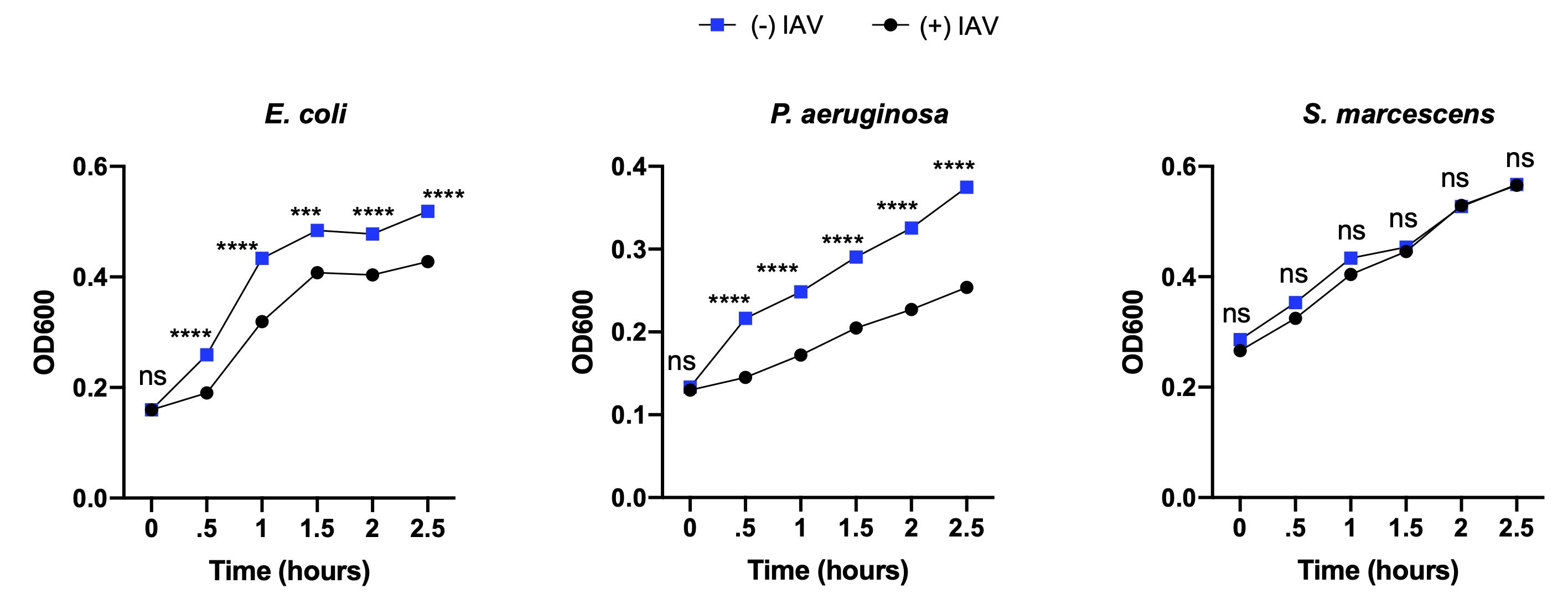

Supplement: S2 Fig — Growth curve of E. coli, P. aeruginosa and S. marcescens in liquid media with/without IAV (PR8). Data points represent mean +/- SD (two-way ANOVA, *p<0.05, **p<0.01, ***p<0.001 and ****p<0.0001). (TIFF) [file ppat.1011020.s002.tiff]

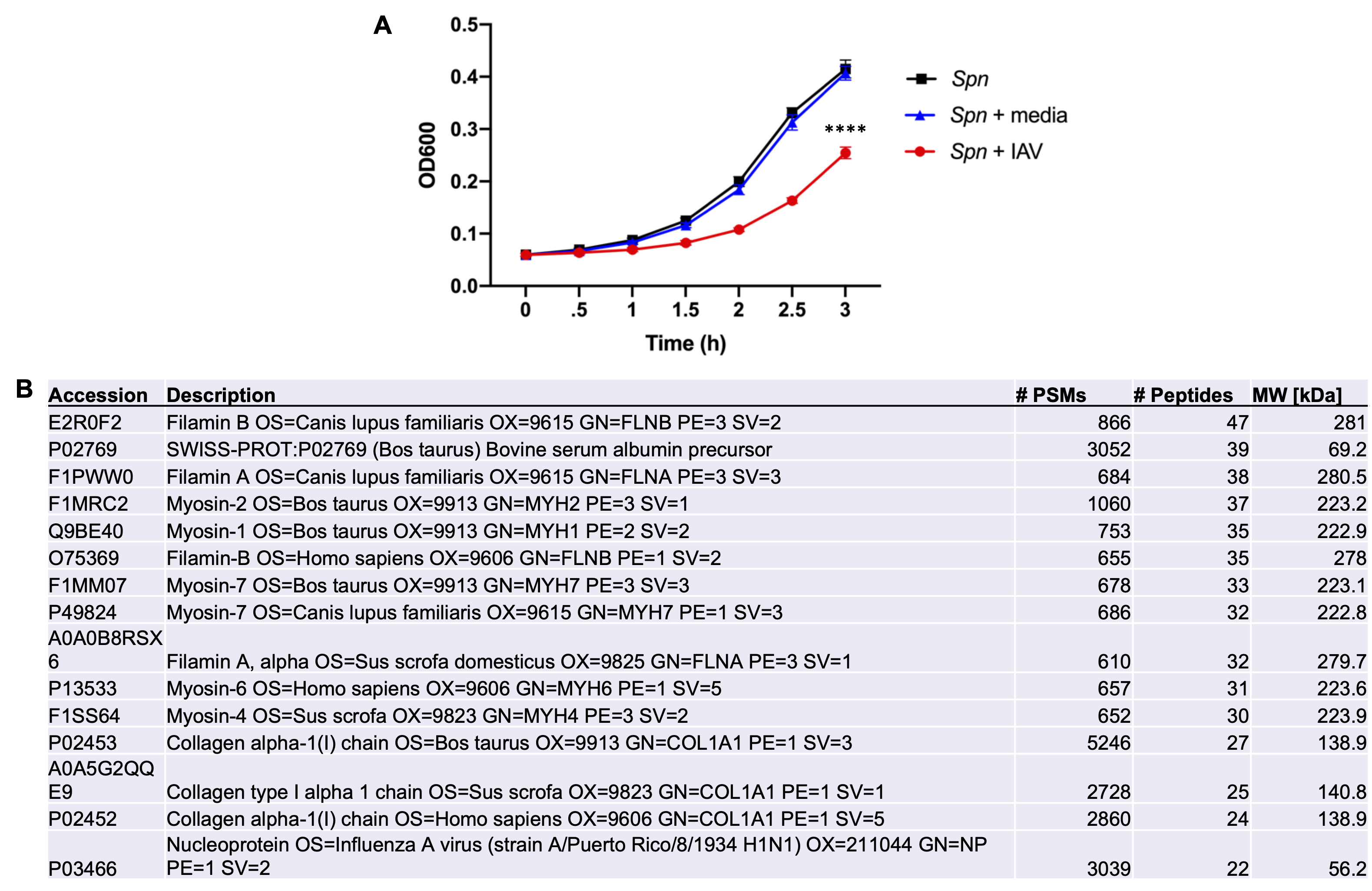

Supplement: S3 Fig — (A) IAV derived from MDCK cells (red circles) blunts Spn growth compared to Spn grown in THY without treatment (black squares), and this effect is independent of cell culture media used in MDCK culture (blue triangles) Data represent n = 8 per condition analyzed by two-way ANOVA, for clarity only significance at 3 h is shown (****p<0.0001). (B) MS analysis of most abundant (top 15) non-bacterial proteins present in the experimental media THY containing influenza A virus. (TIFF) [file ppat.1011020.s003.tiff]

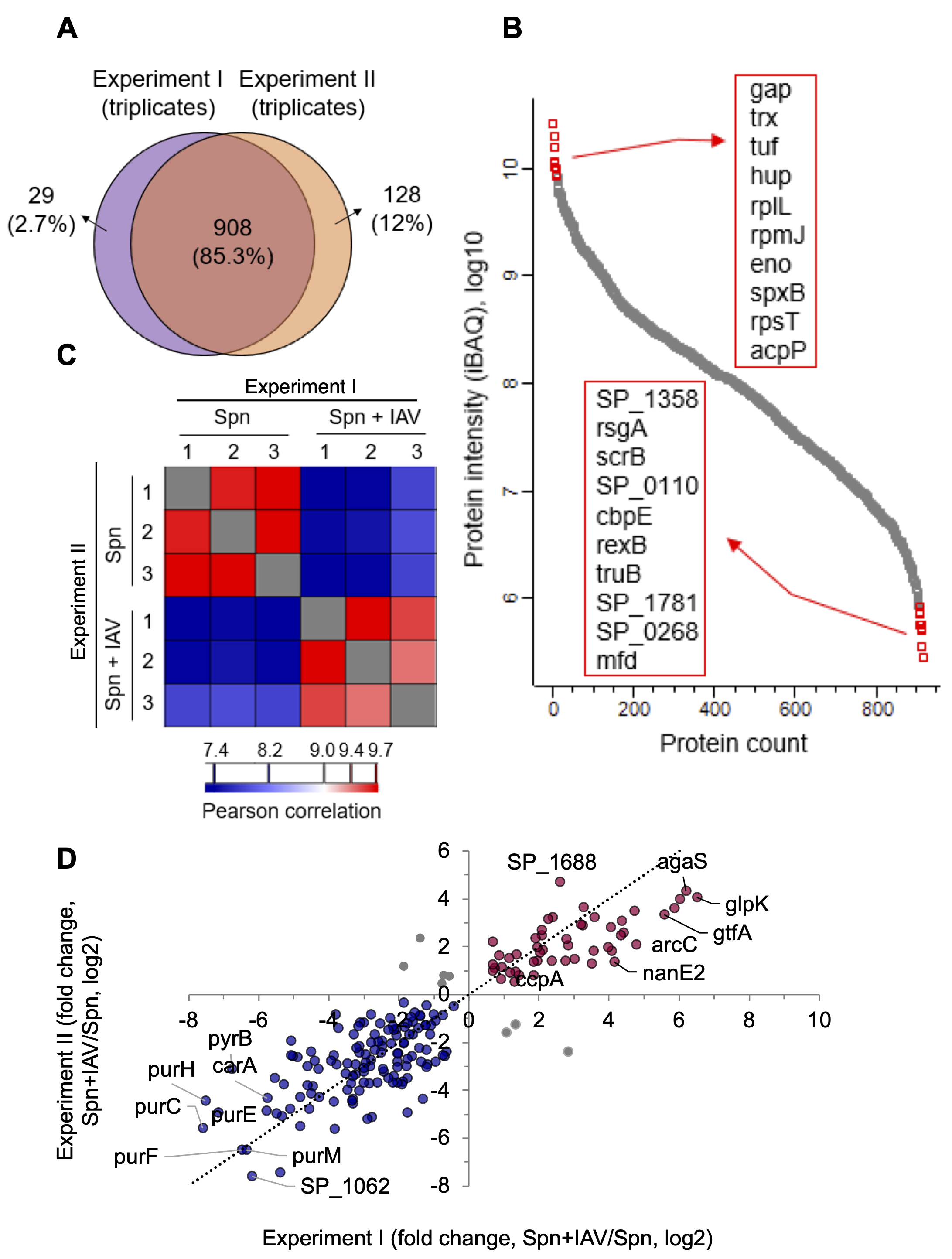

Supplement: S4 Fig — ​ (A) Overlaps between two independent experiments. Each contained three biological replicates. (B) Dynamic range of the quantified Spn proteome. The tope 10 most and least abundant proteins were highlighted in the plot. (C) Pearson correlation among the replicates and the groups. (D) Correlation plot of significantly regulated proteins (p < 0.05) from the two experiments. (TIFF) [file ppat.1011020.s004.tiff]

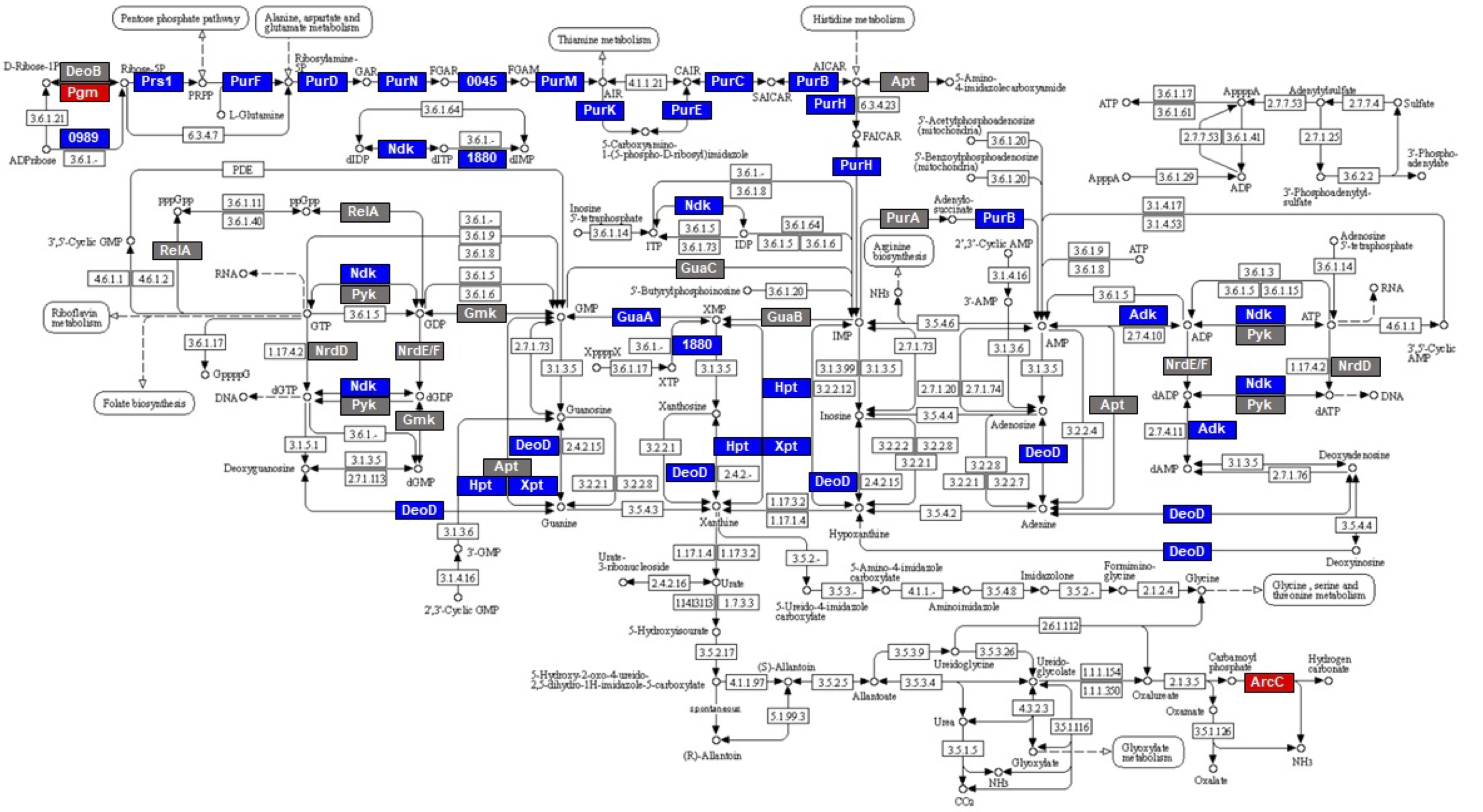

Supplement: S5 Fig — Spn proteins participate in the pathway are in colored box. Red and blue indicates up- or down-regulation when Spn co-incubates with IAV, respectively. Gray indicates no significant changes or not identified in the proteome. (TIFF) [file ppat.1011020.s005.tiff]

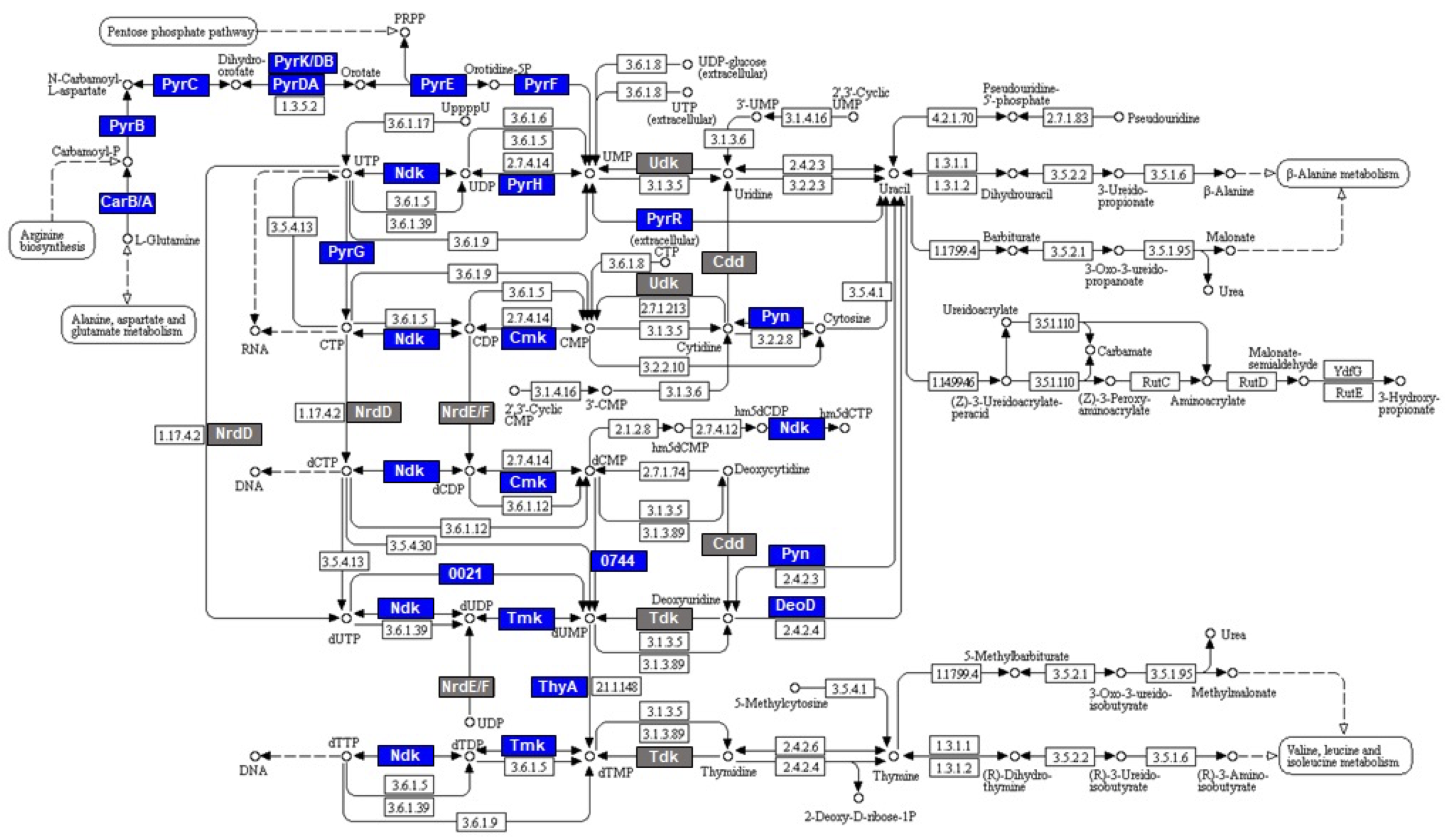

Supplement: S6 Fig — Spn proteins participate in the pathway are in colored box. Red and blue indicates up- or down-regulation when Spn co-incubates with IAV, respectively. Gray indicates no significant changes or not identified in the proteome. (TIFF) [file ppat.1011020.s006.tiff]

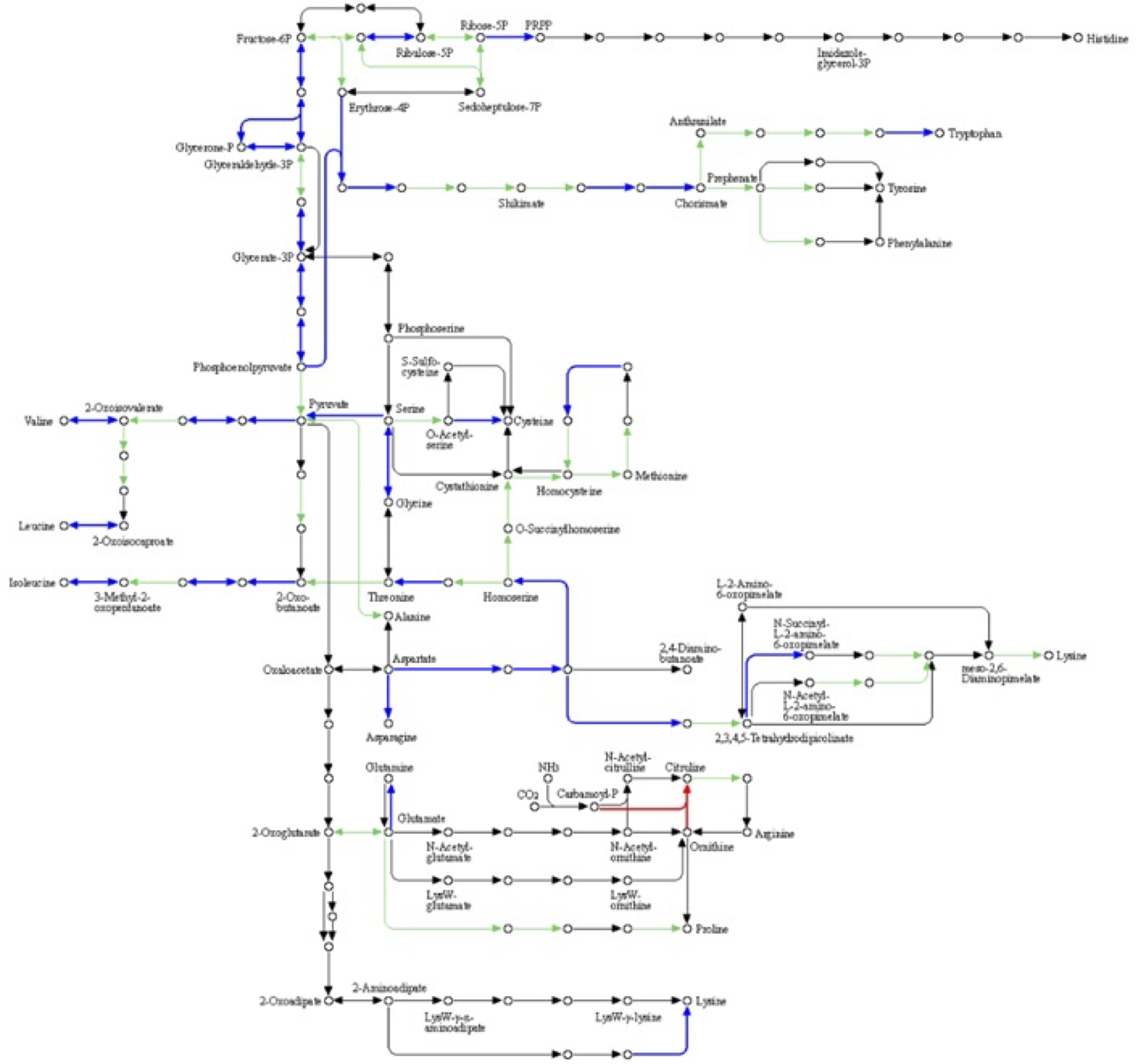

Supplement: S7 Fig — Spn proteins participate in the pathway are in colored lines. Red and blue indicates up- or down-regulation of Spn proteins co-incubated with IAV, respectively. (TIFF) [file ppat.1011020.s007.tiff]

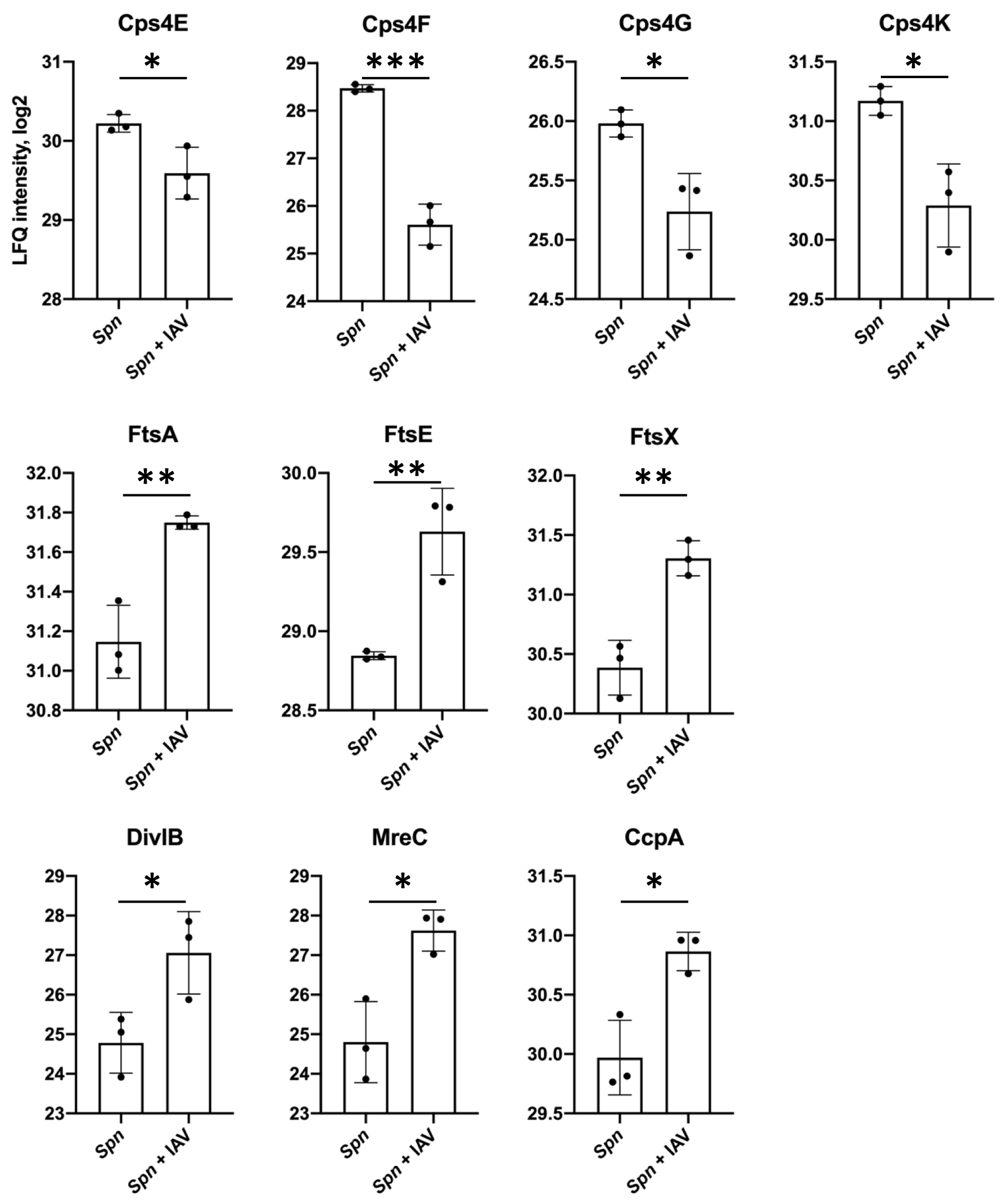

Supplement: S8 Fig — Representative significantly changed Spn proteins were plotted with their protein intensity (quantified by LFQ, log2-transformed) in the presence or absence of IAV. (TIFF) [file ppat.1011020.s008.tiff]

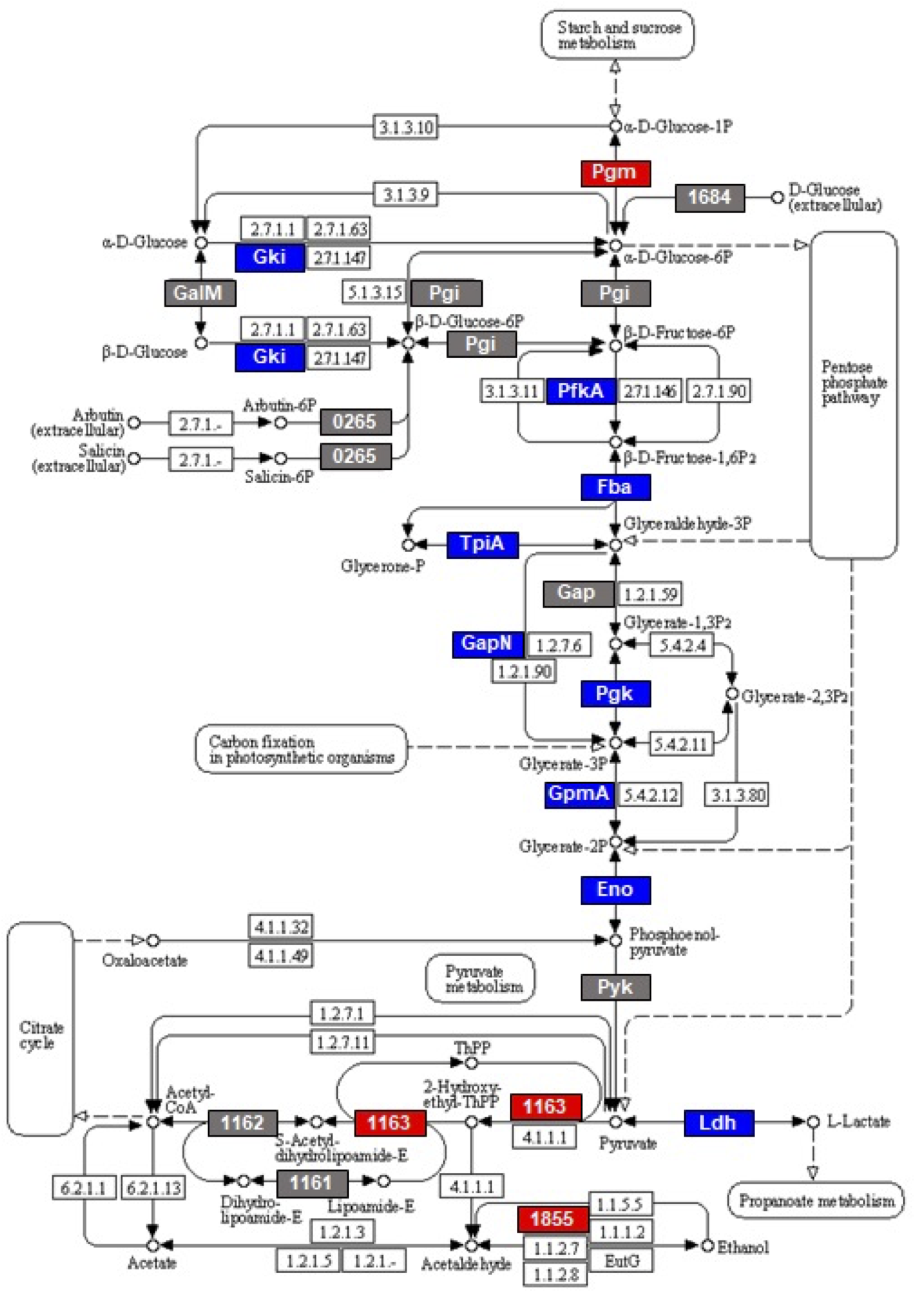

Supplement: S9 Fig — Spn proteins participate in the pathway are in colored box. Red and blue indicates up- or down-regulation when Spn co-incubates with IAV. Gray indicates no significant changes or not identified in the proteome. (TIFF) [file ppat.1011020.s009.tiff]

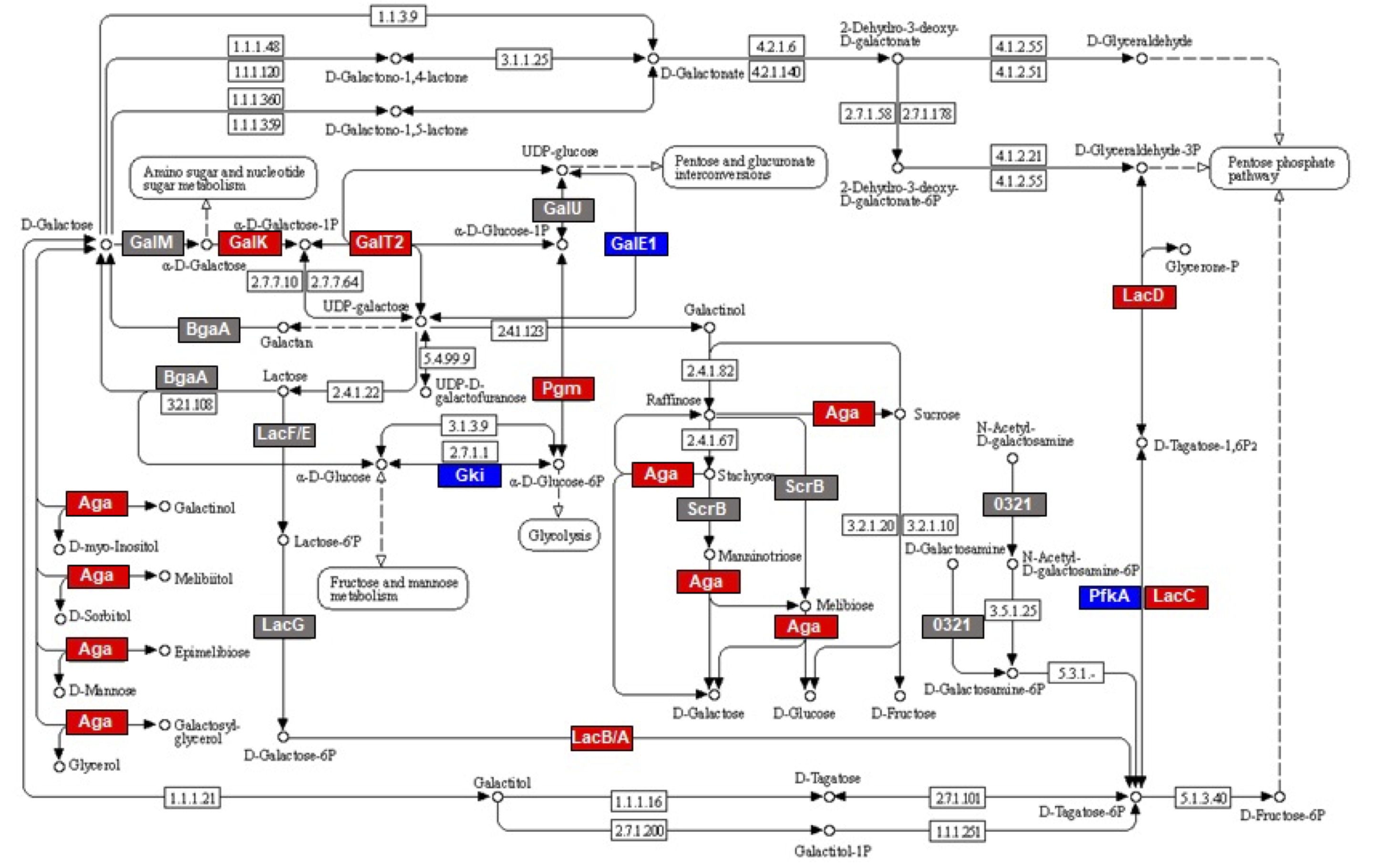

Supplement: S10 Fig — Spn proteins participate in the pathway are in colored box. Red and blue indicates up- or down-regulation when Spn co-incubates with IAV, respectively. Gray indicates no significant changes or not identified in the proteome. (TIFF) [file ppat.1011020.s010.tiff]

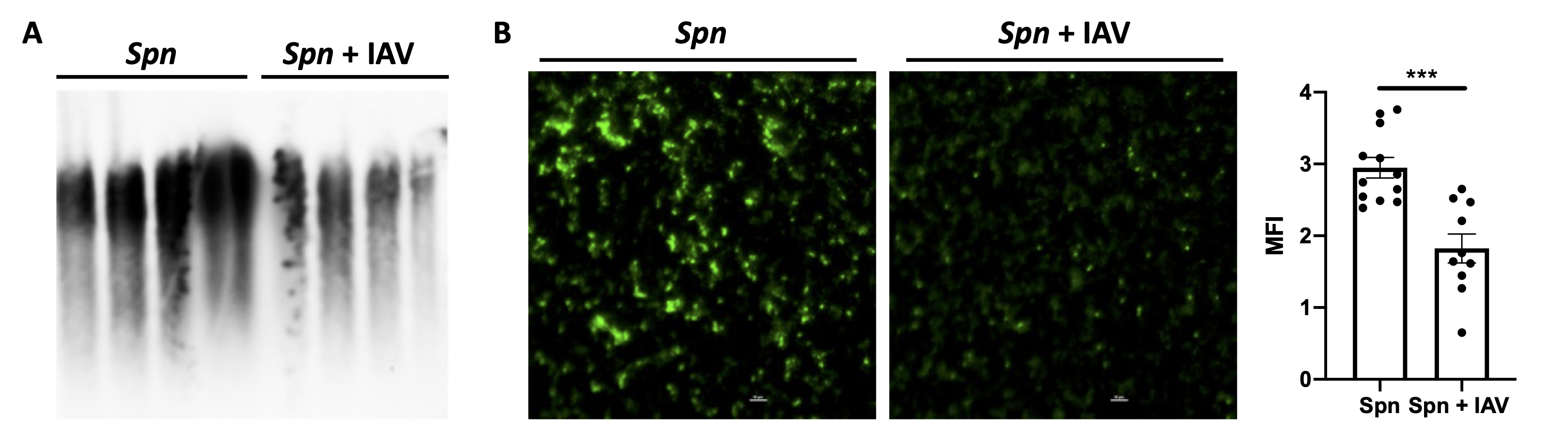

Supplement: S11 Fig — Capsule presence in the bacteria was measured by two methods. (A) Capsule blot from cell lysates and (B) capsule immunofluorescent staining (and mean fluorescent intensity, MFI) of Spn TIGR4 co-incubated with IAV PR8 (1:1) for 1 hour. (TIFF) [file ppat.1011020.s011.tiff]

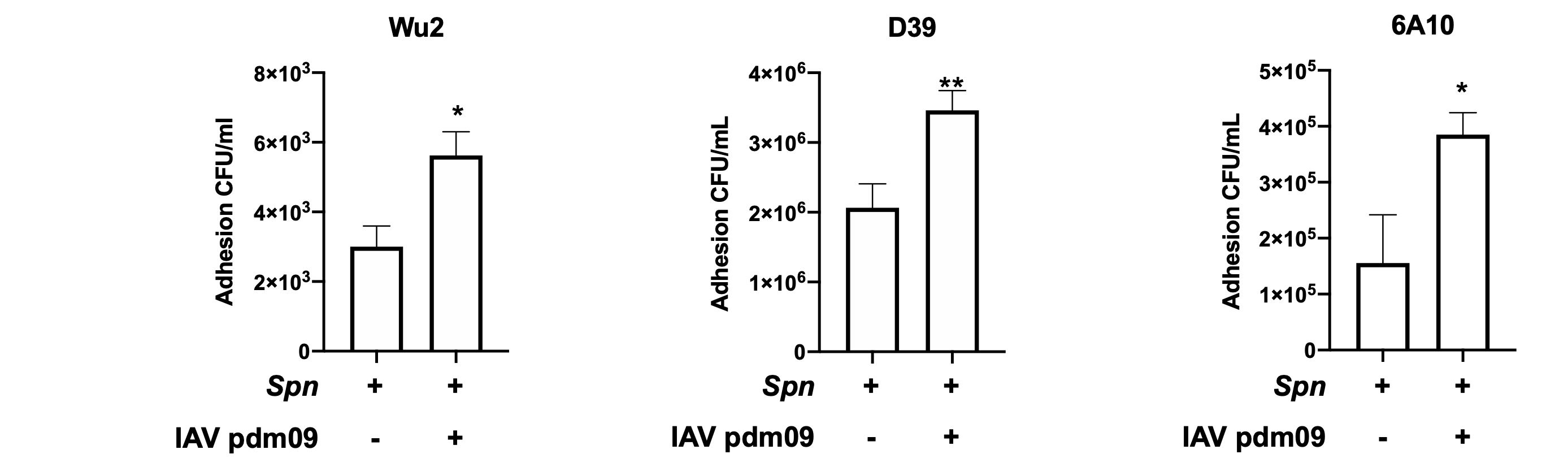

Supplement: S12 Fig — Adhesion assay of Spn serotype WU2, 6A10 and D39 to A549 cells was increased upon incubation with IAV (H1N1 A/California/7/2009 [pdm09]) for 1 hour prior to challenge of cells. Kruskal-Wallis test with Dunn’s multiple comparison post-test. Asterisks denote the level of significance observed: * = p ≤ 0.05; ** = p ≤ 0.01. (TIFF) [file ppat.1011020.s012.tiff]
