# Supplementary material for: A multiomics analysis of direct interkingdom dynamics between influenza A virus and Streptococcus pneumoniae uncovers host-independent changes to bacterial virulence fitness
Source: PLoS Pathog. 2022 Dec 21;18(12):e1011020. doi: 10.1371/journal.ppat.1011020 (PMC9815659; doi:10.1371/journal.ppat.1011020)
Supplement: S1 Table — Spn strain TIGR4 proteins, their gene names are listed. (DOCX) [file ppat.1011020.s013.docx]

**S1 Table. *Spn* surface proteins found to interact with IAV.** *Spn* strain TIGR4 proteins, their gene names are listed.
