# Supplementary material for: A multiomics analysis of direct interkingdom dynamics between influenza A virus and Streptococcus pneumoniae uncovers host-independent changes to bacterial virulence fitness
Source: PLoS Pathog. 2022 Dec 21;18(12):e1011020. doi: 10.1371/journal.ppat.1011020 (PMC9815659; doi:10.1371/journal.ppat.1011020)
Supplement: S3 Table — (DOCX) [file ppat.1011020.s015.docx]

**Name Sequence**

SP16s-1R /5Biosg/GCGTTCTACTTGCATGTATTAGGCACGCCGCCAGCGTTCG

SP16s-2R /5Biosg/TCCATTGCCGAAGATTCCCTACTGCTGCCTCCCGT

SP16s-3R /5Biosg/ACCGCGGCTGCTGGCACGTAGT

SP16s-4R /5Biosg/ACAGCGTGGACTACCAGGGTAT

SP16s-5R /5Biosg/ACCACATGCTCCACCGCTTGTGCGGGCCCCCG

SP16s-6R /5Biosg/ACCCAACATCTCACGACACGAGCTGACGACA

SP16s-7R /5Biosg/GGGCGGTGTGTACAAGGCCCGGGA

SP16s-8R /5Biosg/TTAAGAGATTAGCTTGCCGTCACCGGCTTGC

SP23s-1R /5Biosg/ACCTTTCCCTCACGGTACTGGTTCACTATCGGTCA

SP23s-2R /5Biosg/ACTCGCCGGTTCATTCTACAAAAGGCACGCTCTCACC

SP23s-3R /5Biosg/TCGGAGAGAACCAGCTATCTCCAAGTTCGTTTGGA

SP23s-4R /5Biosg/ATAGCTGCTTCTAAGCTAACATCCTA

SP23s-5R /5Biosg/TAGTACAGGAATATCAACCTGTTGTCCATCGGATACACC

SP23s-6R /5Biosg/TACCTGTGTCGGTTTGCGGTACGGG

SP23s-7R /5Biosg/TCGTGCGGGTCGGAACTTACCCGACAAG

SP23s-8R /5Biosg/GAGCCGACATCGAGGTGCCAAACC

SP23s-9R /5Biosg/CGACGGATAGGGACCGAACTGTCTCACGAC

SP23s-10R /5Biosg/GTGCCAAGGCATCCACCGTGCGCCCT

SP23s-11R /5Biosg/CCACTTCTAACCTATCTACCTGATCATCTCTCAGG

SP5s-1R /5Biosg/GGGTACAGGTGTATCTCCTAGGCTATCGTCAC

SP5S-2R /5Biosg/CTAAGCGACTTCCCTATCTCACAGGGGG

**S3 Table. *rRNA Depletion Primers*.**
